# The genome of the *Lactobacillus sanfranciscensis* temperate phage EV3

**DOI:** 10.1186/1756-0500-6-514

**Published:** 2013-12-05

**Authors:** Matthias A Ehrmann, Angel Angelov, Claudia Picozzi, Roberto Foschino, Rudi F Vogel

**Affiliations:** 1 Lehrstuhl für Technische Mikrobiologie, Technische Universität München, Gregor-Mendel-Str. 4, Freising 85354, Germany; 2 Lehrstuhl für Mikrobiologie, Technische Universität München, Emil-Ramann-Str. 4, Freising 85354, Germany; 3 Dipartimento di Scienze per gli Alimenti, la Nutrizione e l’Ambiente, Università degli Studi di Milano, DeFENS, via Celoria, Milano 2 – 20133, Italy

**Keywords:** Genome, Bacteriophage, EMBL accession number PRJEB61, *Lactobacillus sanfranciscensis*, Sourdough fermentation

## Abstract

**Background:**

Bacteriophages infection modulates microbial consortia and transduction is one of the most important mechanism involved in the bacterial evolution. However, phage contamination brings food fermentations to a halt causing economic setbacks. The number of phage genome sequences of lactic acid bacteria especially of lactobacilli is still limited. We analysed the genome of a temperate phage active on *Lactobacillus sanfranciscensis,* the predominant strain in type I sourdough fermentations.

**Results:**

Sequencing of the DNA of EV3 phage revealed a genome of 34,834 bp and a G + C content of 36.45%. Of the 43 open reading frames (ORFs) identified, all but eight shared homology with other phages of lactobacilli. A similar genomic organization and mosaic pattern of identities align EV3 with the closely related *Lactobacillus vaginalis* ATCC 49540 prophage. Four unknown ORFs that had no homologies in the databases or predicted functions were identified. Notably, EV3 encodes a putative dextranase.

**Conclusions:**

EV3 is the first *L. sanfranciscensis* phage that has been completely sequenced so far.

## Background

In many large-scale food fermentations manufactured with lactobacilli, the risk of bacteriophage contamination is a serious threat. Phage infections are detrimental in industrial dairy or acetic acid fermentations [1-3], where the liquid state of the medium allows the rapid dissemination of the viral particles. Despite spreading of the phage within a sourdough is hindered, probably as a consequence of the semifluid physical state of the matrix, phages of lactobacilli have been already isolated from sourdough samples [4,5] and it has been proven that viral infection can be transmitted from one dough to another [6]. Interestingly, phage spreading into sourdough did neither adversely affect acidification and volume increase of the dough nor reduced lactobacilli cell counts [5].

In a previous work phage EV3 was isolated and phenotypically characterized, showing to be active on five different strains of *L. sanfranciscensis*[5]. This viral particle was ascribed to the *Siphoviridae* family with a morphotype B1. Its lytic life cycle at 25°C lasted 3 h with a burst size of about 30 viral particles per infected cell. The genome estimated by digestion with different restriction enzymes was 31.8 ± 1.5 kbp long, and it was a double-stranded linear DNA molecule with a *pac*-type system. Phage EV3 behaves as a temperate phage that can either multiply via the lytic cycle or enter a dormant state integrating into the host chromosome as a prophage.

Phages may be the most abundant life forms on Earth with a global population on the order of 10^31^[7]. Significant amount of sequencing data is generated by phage genome projects and by sampling of DNA in the environment. Actually, since phages are the main vectors of gene exchange phenomena, they are considered the most important factors in driving evolution in prokaryotes [8].

To date, validated genome sequences of 16 *Lactobacillus* bacteriophages (including prophages) are available from the National Center for Biotechnology Information (NCBI) reference sequence database (RefSeq). The availability of those data allows for comparison of viral genomes in order to understand the genetic relationships among different phages and the function of putative genes. Whereas knowledge on phages and genomes thereof derived from lactic acid bacteria of the dairy environment is increasing, reports on phages coming from cereal fermentations are still rare.

This is the first report of the genome analysis of a *L. sanfranciscensis* phage.

## Results & discussion

### Genome structure

**Figure 1:**
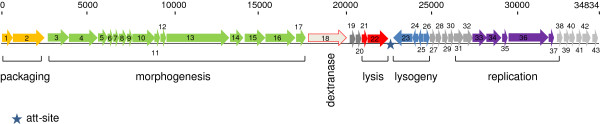
**Map of phage EV3 genome.** Each arrow represents an open reading frame (ORF) and numbering refers to Table 1. Arrows are orientated according to the direction of transcription. The 43 ORFs which were identified are shown, and predicted functions determined by bioinformatic analyses are indicated for the main genes.

EV3 phage belongs to the family of *Siphoviridae* in the order *Caudovirales*. It had a genome length of 34.834 bp with an overall G + C content of 36.45%. Forty-three possible ORFs are numbered consecutively starting from ORF EV3_001 encoding the terminase gene. Amino acids length of ORFs ranged from 51 amino acids (EV_28) to 1263 aa (EV_013). Six of the 43 ORFs were preceded by perfect matching Shine-Dalgarno sequences with the consensus sequence (AGGAGG) that is generally conserved in lactobacilli and was chosen as the recognition sequence for ribosome binding site (RBS) prediction [9]. The consensus sequence is complementary to the 3′ end of the 16S rRNA gene of *L. sanfranciscensis* (5′-CACCTCCTTTCT-3′). Twenty ORFs showed a 1-mismatch RBS and 16 ORFs show less or no sequence similarity. As concern the start codon, ATG predominates (93%). Only ORF EV_15 and the two ORFs EV_34 and EV_40 apparently initiated translation with the TTG start codon and the GTG start codon, respectively (Table 1). A putative function based on similarity level to protein with known functions was assigned to 39 ORFs (Table 1). Highest sequences similarities are with phages infecting lactic acid bacteria. In particular, 13 sequences in the late gene cluster had a similarity with the ones found in *Lactobacillus vaginalis* ATCC 49540 phage whereas six showed correspondence with the ones of *Lactobacillus fructivorans* KCTC 3543 phage. Forty ORFs were oriented in the same direction while three (orf EV3_023, EV3_0 24 and EV3_025) belonging to the lysogeny module were located on the opposite strand. The genome was organized in five functional clusters: DNA packaging, morphogenesis, lysis, lysogeny and DNA replication (Figure 1). Between morphogenesis and lysis clusters there was a peculiar ORF coding for a dextranase.

**Table 1:** **Open reading frames and genetic features of *L. sanfranciscensis *** **EV3 phage**

**Locus tag**	Putative RBS^* ^**and start codon**^**‡**^	**nt**	**orf length (aa)**	**Best hit**	**Best hit EMBL protein name**	**Score**
EV3_001	ttacgaa*aggaga*aattgt**atg**	136-675	179	Phage terminase *L. vaginalis,* small subunit ATCC 49540	EEJ41449	1.0E-88
EV3_002	gacagtattgctaatatgctaa**atg**	650-2590	646	Phage terminase *L. vaginalis*, large subunit, ATCC 49540	EEJ41450.1	0.0
EV3_003	tgcgat*aggagg*tgattagca**atg**	2786-3964	398	Phage portal protein *L. vaginalis* ATCC 49540	EEJ41452.1	0.0
EV3_004	taa*aggagg*tgataatgtaa**atg**	3930-5918	662	phage capsid protein *L. vaginalis* ATCC 49540	EEJ41453.1	0.0
EV3_005	caaaataa*aggagt*gataaa**atg**	5950-6489	179	major tail protein *Staph. pseudintermedius* HKU10-03	ADV05789.1	1.0E-13
EV3_006	actaa*gggaga*tgagtagca**atg**	6509-6814	101	Putative uncharacterized protein *L. vaginalis* ATCC 49540	EEJ41454.1	6.0E-45
EV3_007	aacaagggtggtgacagcca**atg**	6808-7176	122	Head-tail joining protein *L. vaginalis* ATCC 49540	EEJ41455.1	5.0E-155
EV3_008	tgtaa*aggagc*tgagtggca**atg**	7169-7606	145	Head-tail joining protein *L. vaginalis* ATCC 49540	EEJ41456.1	2.0E-91
EV3_009	aa*aggatg*ttaaatcatgaaa**atg**	7603-7992	129	Phage tail protein *L. vaginalis* ATCC 49540	EEJ41424.2	7.0E-66
EV3_010	ttagaa*aagagg*aaataattt**atg**	7996-8763	255	Phage major tail protein *L. vaginalis* ATCC 49540	EEJ41425.1	1.0E-123
EV3_011	cggccatta*aggaga*taagta**atg**	8873-9283	136	Putative uncharacterized protein *L. vaginalis* ATCC 49540	EEJ41426.1	9.0E-64
EV3_012	gtcgtaagccaaacgtggctctg**atg**	9343-9513	56	Putative uncharacterized protein *L. vaginalis* ATCC 49540	EEJ41427.1	7.0E-22
EV3_013	gaagga*aggagg*taactaa**atg**	9514-13305	1263	Phage minor tail protein *L. vaginalis* ATCC 49540	EEJ41428.1	0.0
EV3_014	aacttaat*ggagg*tcttgcata**atg**	13305-14141	278	Putative uncharacterized protein *L. saliv*arius (strain CECT 5713)	ADJ78578.1	3.0E-48
EV3_015	aattttag*ggagg*tgttaat**ttg**	14161-15396	411	Glycosylhydrolase *L. plantarum*	CCC78574.1	4.0E-17
EV3_016	aggaacaagggtgattattta**atg**	15396-17177	593	Put. Minor structural protein *Leu. kimchii* IMSNU 11154	ADG39890.1	3.0E-62
EV3_017	tttgtctag*gaagg*agaaaaa**atg**	17190-17726	54	Hypothetical protein	No hit	-
EV3_018	aaaagttgggagtgattaaaa**atg**	17729-20134	801	Dextranase *L.* f*ermentum* phage phiPYB5	ADA798961.1	1.0E-137
EV3_019	atgaaataaa*ggaga*aaataa**atg**	20212-20721	170	hypothetical protein	No hit	-
EV3_020	tggagaaa*ggagg*tgatgaa**atg**	20790-21083	97	Predicted protein *P. acidilactici*	EFA25777.1	2.0E-4
EV3_021	aaagaaacgagtgaacaat**atg**	21067-21309	80	*L. plantarum* WCFS1 phage P1 holin, lp0683	F9ULS1	6.0E-68
EV3_022	taccattaatcatcgttgctgaa**atg**	21383-22429	348	putative endolysin *L. vaginalis* ATCC 49540	EEJ39754.1	9.0E-70
EV3_023	aaagcagaaagcgaattaat**atg**	23961-22876c	361	phage integrase ***L. salivarius *ACS-116-V-Col5a**	EFK79983.1	1.0E-112
EV3_024	atattctaa*ggaga*atggaaa**atg**	24072-24485c	137	Putative uncharacterized protein *L. pentosus* MP-10	CCB81756.1	4.0E-14
EV3_025	tagaatacgattggatattgat**atg**	24478-24795c	110	XRE family transcriptional regulator L. pentosus	FR871768	4.0E-23
EV3_026	tatgaaaa*ggagg*aatcaat**atg**	25072-25263	63	Phage antirepressor *L. ruminis* ATCC 25644	EFZ34631.1	2.0E-10
EV3_027	agaaaaaagatgattgtc**atg**	25378-25638	89	Phage protein *Listeria monocytogenes* FSL N1-017 helix-turn-helix protein	EFK40475.1	4.0E-5
EV3_028	tata*aggggg*tgagatag**atg**	25639-25794	51	Hypothetical protein	No hit	-
EV3_29	atttca*aggaga*tgtaataa**atg**	25798-26028	77	Hypothetical protein	No hit	-
EV3_030	aatctaaa*aggaag*ttattcat**atg**	26146-26322	59	hydrolase NUDIX family *L. delbrueckii*	CAI96847	0.73
EV3_031	aattttagt*ggggg*tagagaa**atg**	26336-26821	161	Putative uncharacterized protein *Mahella australiensis* DSM 15567	AEE95754.1	6.0E-7
EV3_032	ccgaaa*ggaag*tgagataa**atg**	26852-27319	155	Putative uncharacterized protein lp_0862 *L. pentosus* IG1	FR874854.1	1.0E-22
EV3_033	actaaattata*ggaga*taaat**atg**	27380-28120	246	NTP-binding protein *L. paracasei subsp. paracasei* ATCC 25302	EEI67797.1	5.0E-71
EV3_034	agtttggaagtgatgaaaacg**gtg**	28068-29459	463	Putative helicase *Lactobacilus* phage A2	CAB63670.1	1.0E-130
EV3_035	acaaata*ggaga*aaaatatt**atg**	29479-30042	187	Single stranded binding protein *L. hilgardii* ATCC 8290	EEI25831.1	4.0E-41
EV3_036	aatatatgaaagggaaaattt**atg**	30115-32442	775	Phage primase, P4 family *L. buchneri* NRRL B-30929	AEB73788.1	0.0
EV3_037	taattttaa*ggagg*aacacaa**atg**	32745-33152	135	Hypothetical protein	No hit	-
EV3_038	atgaaagatgtgatgtctgata**atg**	33145-33465	106	VRR-NUC domain Phage protein *L. plantarum* JDM1	ACT61379.1	2.0E-33
EV3_039	tgaatatagta*ggag*catttaa**atg**	33462-33686	75	Hypothetical protein	No hit	-
EV3_040	aagattgggagaaaataacc**gtg**	33676-33849	58	Hypothetical protein	No hit	-
EV3_041	caatgtaa*ggaag*aatgata**atg**	33872-34114	80	Ribonucleoside-diphosphate reductase 2, *Ent. faecalis*	AAO80328.1	3.0E-13
EV3_042	ataatcgtttcgttgggggttatt**atg**	34196-34612	138	Phage transcriptional regulator *Lactobacillus* phage phig1e	CAA66778.1	3.0E-14
EV3_043	taaacaaaa*ggagt*agttaat**atg**	34666-34833	56	Putative transporter protein *L. reuteri* ATCC 53608	CCC04545.1	3.0E-14

*Putative ribosomal binding sites are printed in italics.

^‡^Putative start codons are printed in bold.

### EV3 DNA packaging

The predicted protein products of ORF EV3_01 and EV3_02 were similar to the putative small and large terminase subunits from *L. vaginalis* ATCC 49540 phage. In tailed phages, terminases consist of a large subunit containing the ATPase activity that controls DNA translocation together with an endonuclease activity that cuts concatemeric DNA into genome lengths, and a small subunit responsible for specific DNA binding. Therefore, these two EV3 proteins were probably involved in DNA packaging. In a previous work [5] it was already highlighted that EV3 had no *cos* site and therefore it is likely to pack its DNA through a *pac* system. The protein encoded by ORF EV3_035 had a high similarity with the putative DNA binding protein of *L. hilgardii* ATCC 8290. Its position was quite close to terminases genes suggesting that the putative gene product of ORF EV3_035 could also be involved in DNA packaging.

### DNA morphogenesis

ORF EV3_003 and EV3_004 constituted the putative head module, since they were similar to portal protein and capsid protein of *L. vaginalis* ATCC 49540 phage*,* respectively. The portal complex forms a channel through which the viral DNA is packaged into the capsid, and exits during infection. The portal protein is thought to rotate during DNA packaging. It also forms the junction between the phage head (capsid) and the tail proteins. Putative gene products encoded by ORF EV3_007 and EV3_008 were likely to connect head and tail structures. The overlapping of the two genes suggest a translational coupling. The putative tail module was positioned downstream from the predicted head-tail-joining genes, and it was composed by ORF EV3_009, EV3_010. EV3_013 encoded product was similar to various tail component and tape measure proteins (TMP) from phages of *L. vaginalis and L. fermentum*. TMP generally works as template for measuring length during tail assembly, thus, it is reasonable to ascribe this function to the protein.

### Lysis module

The predicted protein product from ORF EV3_021 had a 44% overall identity with the holin of *L. plantarum* WCFS1 phage P1. Holins are a diverse family of proteins that cause bacterial membrane lysis during late-protein synthesis. ORF EV3_022 encodes a putative endolysin that is quite similar (54% identity) to the endolysin of *L. vaginalis* ATCC 49540. The C terminus of this ORF contains two Lysine Motif domains that are likely to be implicated in bacterial cell wall degradation, while the N terminus encloses a Cpl-1 lysin (also known as Cpl-9 lysozyme/muramidase) that is a bacterial cell wall endolysin. A signal peptide with a predicted cleavage site (probability of 0.750) between position 26 and 27 of the amino acid sequence was identified. An analogous signal peptide was already reported for other phages of lactic acid bacteria and was demonstrated to be active [10,11].

### Integrase module and attachment site

ORF EV3_023 has an amino acid sequence comparable to phage integrase of *L. salivarius* ACS-116-V-Col5a phage. In order to identify the attP site the non coding sequence of 429 bp between the lys (orf EV3_22) and int (orf EV3_23) genes of the phage EV3 was blasted against the whole genome sequence of *L. sanfranciscensis* TMW 1.1304 the only strain whose genome sequence is available [12]. We found only one significant hit of 16 nucleotides matching the 3′ end of a tRNA_Leu_ gene. Since the host attachment sites are commonly located near tRNA genes [13], we assumed this sequence as putative attB site in *L. sanfranciscensis* H2A.

**Figure 2:**
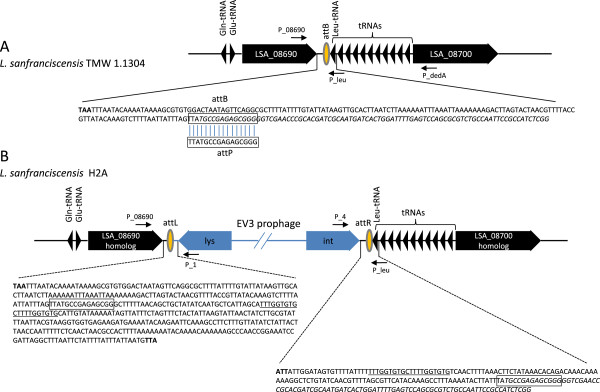
**Schematic representation of the bacterial chromosomal attachment site (attB) of *L. sanfranciscensis *TMW 1.1304 (A) and prophage integration in strain H2A (B) tRNAleu sequence is italicized.** Only the outmost prophage lysin and integrase genes are depicted. The arrows provide the locations of the primers used for PCR. Relevant nucleotide sequences are provided in enlarged inserts. Stop codons of flanking genes are in bold. Putative att sites are boxed. Repeats are underlinded.

By using primers placed in the bacterial genes flanking the prophage in combination with primers whose binding sites are placed within the int and lys gene, respectively we amplified 658 bp and 370 bp long DNA fragments when DNA of *L. sanfranciscensis* H2A carrying EV3 as a temperate phage was used as template. Sequence analysis revealed that indeed prophage EV3 is located between the putative orf LSA_08690 on the *L. sanfranciscensis* genome map on one side and a tRNA_Leu_ gene on the other (Figure 2). The deduced attB were identical to those to the left and the right, respectively, thus identifying them as the attL and attR sites. Additionally, EV3 is flanked by two 20-bp repeats suggesting a Campbell-like integration of prophage EV3 into the tRNA_Leu_ gene that is functionally reconstituted upon prophage integration. Other *L. sanfranciscensis* strains from our collection amplified a 314 bp PCR product when primers were placed in the bacterial genes that bracket the prophage in H2A (data not shown).

Most probably the sequence: 5′ GCCGAGAGCGGG 3′ found on *L. sanfranciscensis* genome, is the region recognised by the bacteriophage (*att*B site) since an homologous region was found also on EV3 genome (*att*P site). The *att*B region, located between the Lysin and the Integrase genes in lactobacilli containing the phage, corresponds to a gene encoding for a tRNA confirming that some phages integrate their genome directly into genes for the tRNA.

### Lysogeny

The protein encoded by ORF EV3_025 is similar to XRE family transcriptional regulator of *L. pentosus* MP-10. This large family of DNA binding helix-turn helix proteins includes Cro and CI. The product encoded by EV3_026 shows an identity with phage antirepressor of *L. vaginalis* ATCC 49540 phage. This protein is thought to promote transcription of genes required for phage production.

### Phage replication module

ORF EV3_027 had a DNA binding domain in the N-terminal region with an identity to excisionase protein (Xis protein) and a helix-turn-helix (HTH) DNA binding domain. The predicted proteins from EV3_028 to EV3_032 have an unknown function or they are not characterized. ORF EV3_033 and EV3_034 encode for a phage-DNA binding protein and a helicase, respectively.

### Dextranase gene

The gene is active since it was experimentally shown that clones of H2A strain hosting EV3 phage become dextranase positive [14]. To our knowledge this is the second time that a gene encoding for this enzyme has been found in the phage genome of a lactobacillus [15]. Looking at the position of dextranase gene in the sequences of the viral genome we could speculate that such enzymatic activity can help the viral particle in breaking through dextran producing strains after cell lysis occurs.

Temperate phages are known to carry virulence genes that contribute to the “success” of pathogenic bacteria. There is also a substantial scientific literature explaining this phenomenon by evolutionary arguments [16]. It could well be that temperate phages play this role only for pathogenic bacteria. However, some theoretical reasoning suggests that prophages from non-pathogenic bacteria should encode more general fitness genes that are of selective benefit to the lysogen and/or the host, albeit up to know there is no direct evidence demonstrating prophages encoded fitness factors on of bacterial commensals or food microbes. The present manuscript may be one of the best hints so far for such a fitness factor in the field of LAB, which comprise many industrially important food bacteria. The demonstration of such a fitness factor could thus have an important impact on theoretical reasoning about the role of phages for the evolution of bacteria in general.

### Phyogeny

The phylogenetic position of EV3 was evaluated using the large subunit of the terminase gene as well as the large subunit portal protein gene. These genes have been previously established as valuable marker for phage phylogeny [17,18]. Both marker genes positioned EV3 to a monophyletic group together with phages identified in the genomes of *L. vaginalis, L. fermentum, L. jensenii, L. rhamnosus and L. casei*. These species are neither the closest relatives to *L. sanfranciscensis* nor typically isolated from the sourdough ecosystem. This result may be reflected by the current unavailability of genome sequences of lactobacilli adapted to sourdough fermentations.

## Conclusions

To our knowledge, this study represents the first complete genome sequence and genetic characterization of a *L. sanfranciscensis* phage. Bioinformatic analysis revealed that phage EV3 is a unique temperate phage compared to phages infecting related species of LAB. The endolysin gene was preceeded by a holin gene. The tail morphogenesis module is interspersed with cell lysis genes. The overall amino acid sequences of the phage proteins had little similarity to other sequenced phages. The phage carries a dextranase gene whose function in establishing a stable relationship with their host (lysogen) and influencing its lifestyle and fitness in sourdough fermentations remains to be elucidated. The results of this study may provide new insights that deepen our understanding of phage genetics and phage-host interactions in dynamic ecosystem such as cereal fermentations.

### Availability of supporting data

The phage EV3 genome sequence is deposited at EMBL accession number PRJEB61 http://www.ebi.ac.uk/ena/data/view/display=html&PRJEB61.

## Methods

### Isolation of phage DNA

*L. sanfranciscensis* H2A strain was used as host culture for viral multiplication. Phage DNA was isolated from a high-titer phage lysate obtained by cesium chloride gradient according to Sambrock et al., [19].

### Sequencing strategy

For full sequencing, purified phage DNA was fragmented by ultrasonification, and ligated into the plasmids pBluescriptKSII and pSmart. Escherichia coli DH5a cells were transformed and colonies were selected by blue/white selection.

Sequencing was performed on 3 × 96 shotgun clones by Sanger sequencing, resulting in a sixfold genome coverage.

Remaining gaps were closed by a Two-Step Gene Walking technique based on randomly primed polymerase chain reaction (PCR) as previously described by Pilhofer et al., [20]. Amplification were performed by use of Kapa2G-Robust Polymerase (Kapa Biosystems, Inc.). It presents a simple workflow, which comprises only two major steps of a Walking-PCR with a single specific outward pointing primer (step 1) and the direct sequencing of its product using a nested specific primer (step 2). Open reading frames (ORFs) were predicted with Gene- Mark.hmm for Prokaryotes, Version 2.4 [21]. All ORF predictions were verified and modified by blasting ORFs to NCBI nrdb. Additionally, the predicted start codons of all ORFs were inspected manually using the Artemis program [22]. This genome project has been deposited in the European Molecular Biology Laboratory (EMBL)/Gen- Bank under the accession number PRJEB61. The presence of signal peptides was analysed with SignalP (http://www.cbs.dtu.dk/services/SignalP/).

### Determination of the attachment site on the host genome

In order to identify the attP site we blasted the sequence of 429 bp between the lys and int genes of the phage EV3 against the whole genome sequence of *L. sanfranciscensis* TMW 1.1304 [12].

Primers P_08960 (5′-ATGGAAAAATCGATGTATG) and P_leu (5´-GCCGAGATGGCGGAATTG) placed in the bacterial genes flanking the prophage (orf LSA_08960 and Leu-tRNA), and primers P4 (5′-CGTCGATATTTATATCATTAG) and P1 (5´-GATACCTTAACCAGATTAAG) running out of the int and lys genes we amplified 658 bp and 370 bp long DNA fragments, respectively (Figure 2).

## Competing interests

The authors declare that they have no competing interests.

## Authors’ contributions

MAE carried out the molecular genetic studies, participated in the genome sequencing and drafted the manuscript. RFV participated in its design and coordination and participated in drafting the manuscript. AA performed the genome annotation. CP participated in the design of the study and drafting the manuscript. RF conceived of the study, and helped to draft the manuscript. All authors read and approved the final manuscript.
